# Selection bias in multidrug-resistant tuberculosis cohort studies assessing sputum culture conversion

**DOI:** 10.1371/journal.pone.0276457

**Published:** 2022-11-10

**Authors:** Carly A. Rodriguez, Sara Lodi, C. Robert Horsburgh, Mathieu Bastard, Cathy Hewison, Helena Huerga, Munira Khan, Palwasha Y. Khan, Uzma Khan, Lawrence Oyewusi, Shrivani Padayachee, Carole D. Mitnick, Molly F. Franke

**Affiliations:** 1 Department of Epidemiology, Boston University School of Public Health, Boston, Massachusetts, United States of America; 2 Department of Global Health and Social Medicine, Harvard Medical School, Boston, Massachusetts, United States of America; 3 Department of Biostatistics, Boston University School of Public Health, Boston, Massachusetts, United States of America; 4 Epicentre, Paris, France; 5 Médecins Sans Frontières, Paris, France; 6 THINK TB & HIV Investigative Network, Durban, South Africa; 7 Interactive Research and Development Global, Singapore, Singapore; 8 Department of Clinical Research, London School of Hygiene and Tropical Medicine, London, United Kingdom; 9 Partners In Health, Maseru, Lesotho; Texas A&M University College Station, UNITED STATES

## Abstract

**Background:**

Conversion of sputum culture from positive to negative for *M*. *tuberculosis* is a key indicator of treatment response. An initial positive culture is a pre-requisite to observe conversion. Consequently, patients with a missing or negative initial culture are excluded from analyses of conversion outcomes. To identify the initial, or “baseline” culture, researchers must define a sample collection interval. An interval extending past treatment initiation can increase sample size but may introduce selection bias because patients without a positive pre-treatment culture must survive and remain in care to have a culture in the post-treatment interval.

**Methods:**

We used simulated data and data from the endTB observational cohort to investigate the potential for bias when extending baseline culture intervals past treatment initiation. We evaluated bias in the proportion with six-month conversion.

**Results:**

In simulation studies, the potential for bias depended on the proportion of patients missing a pre-treatment culture, proportion with conversion, proportion culture positive at treatment initiation, and proportion of patients missing a pre-treatment culture who would have been observed to be culture positive, had they had a culture. In observational data, the maximum potential for bias when reporting the proportion with conversion reached five percentage points in some sites.

**Conclusion:**

Extending the allowable baseline interval past treatment initiation may introduce selection bias. If investigators choose to extend the baseline collection interval past treatment initiation, the proportion missing a pre-treatment culture and the number of deaths and losses to follow up during the post-treatment allowable interval should be clearly enumerated.

## Background

During tuberculosis (TB) treatment, particularly drug-resistant TB (DR-TB), sputum cultures are routinely monitored for growth of *Mycobacterium tuberculosis* (*M*.*tb*). Conversion of culture from positive to negative for *M*.*tb* is an important sign of treatment response and is often used as an early indicator of treatment outcome [1–4]. Although the World Health Organization (WHO) recommends monthly culture monitoring [5], in observational TB cohorts treated under programmatic conditions, patient encounters and sample collection may occur less frequently, and limited laboratory services or reagent stock-outs can result in inconsistent data.

An initial, “baseline” positive culture is a pre-requisite to observe conversion to negative culture. Only patients with positive cultures at a pre-defined baseline time point (generally before treatment initiation) are included in conversion analyses; patients with a negative baseline culture or a missing baseline culture are excluded. Under programmatic conditions, patients may not have a culture result immediately before treatment initiation or may not have a culture result before treatment initiation at all. Therefore, investigators will define an interval before treatment initiation constituting the baseline culture. In some cases, investigators may extend this interval past treatment initiation to include patients who lacked a pre-treatment culture but had a culture after treatment initiation [6]. While the latter may improve precision by increasing sample size, it could also introduce selection bias. This is because the analysis cohort will include those without a positive pre-treatment culture who survived or were retained long enough to have a recorded positive culture in the post-treatment interval, but exclude those without a positive pre-treatment culture who die or are lost to follow up (LTFU) during this interval, events often defined as non-conversions. Inclusion in the study requires a subset of patients (i.e. patients missing a pre-treatment culture) to survive or be retained in the study long enough to make it past a selection process (i.e. having a culture in the post-treatment initiation interval) [7, 8]. The ramifications of selection bias have been assessed at length in the epidemiologic literature [9–15].

Using simulations and observational data from a cohort of DR-TB patients, we investigate the potential for bias when reporting the proportion of a cohort with culture conversion when the baseline culture collection interval is extended past treatment initiation.

## Methods

### Quantifying bias using simulated data

We first simulated a hypothetical cohort with complete pre-treatment culture data and then introduced missing data under different scenarios to explore its impact on conversion. Conducting bias analyses in real-world data is limited by the range of values observed in the dataset. By simulating data, we can generate the full range of potential values for parameters relevant to conversion, in order to better understand the scenarios where bias can occur [16, 17]. To simulate a cohort, we used a tree graph to define the subsets of patients in a given hypothetical TB cohort, which is detailed in **Supporting Information**. Using the tree graph as a guide, we inferred an equation to quantify the difference in the true and observed proportions with culture conversion, resulting in the parameters specified below. The values applied to each of the following parameters are listed in Table 1.

**Table 1:** Parameters and values, simulation study of bias due to early death and loss-to-follow up events occurring during a hypothetical post-treatment initiation sputum collection interval among participants missing a pre-treatment sputum culture.

Parameter (abbreviation)	Description	Simulated values
*Culture positive*_*truth*_ (P_t_)	Proportion of patients who would have been observed to be culture positive at the time of treatment initiation, had they had a sputum culture	60% to 90% by 10%
*Culture missing*_*observed*,_ (m)	Proportion of patients missing a pre-treatment culture	0% to 30% by 5%
*Culture positive*_*truth*_*│Culture missing*_*observed*_ (P_t_│m)	Proportion of patients with a missing pre-treatment culture, who would have been observed to have a positive culture at treatment initiation, had they had a culture	0% to 100% by 25%
*Converted│Culture positive*_*truth*_ (C│P_t_)	Proportion of patients with conversion among patients who would have been observed to have a positive culture at the time of treatment initiation, had they had a sputum culture	50% to 90% by 20%

*Culture positive*_*truth*_ (abbreviated P_t_), represents the proportion who would have been observed to be culture positive at the time of treatment initiation, had they had a culture.

*Culture missing*_*observed*_ (abbreviated m), represents the proportion missing a pre-treatment culture.

*Culture positive*_*truth*_*│Culture missing*_*observed*_ (abbreviated P_t_│m), represents the proportion with a missing pre-treatment culture, who would have been observed to have a positive culture at treatment initiation, had they had a culture.

*Converted│Culture positive*_*truth*_ (abbreviated C│P_t_), represents the proportion with conversion among patients who would have been observed to have a positive culture at the time of treatment initiation, had they had a culture.

The proportion with conversion is calculated by dividing the number of patients observed to have converted by the number of patients observed with a positive baseline culture. We calculate the observed proportion with conversion (*Converted│Culture positive*_*observed*_ (abbreviated C│P_O_)) as follows: $\frac{P_{t}\;\times {\;C│P}_{t}\;}{P_{t}\;-\;(m\;\times {\;P}_{t}│m)}$ (Supporting Information). This formula includes all conversion events in the numerator and subtracts from the denominator patients missing a culture who would have been positive, had they had a culture in the interval. We hypothesized early deaths and LTFU occurring during the post-treatment interval would drive differences between true and observed conversion frequencies, and therefore that conversion in patients missing a pre-treatment culture occurred at an equal or lower frequency than among patients observed to have a pre-treatment culture. This assumption limits C│P_O_ to values greater than or equal to C│P_t_, We report the difference between C│P_t_ and C│P_O_ and the minimum and maximum proportion of the cohort observed to have converted and percentage point discrepancy between the two figures, where the maximum proportion observed to have converted assumes all patients with P_t_│m = 1 do not convert. Values presented in Table 1 were simulated irrespective of whether, in combination, they produced results that exceeded the 0.00 to 1.00 bounds of a proportion (e.g., 0.70 * 0.80 / [0.70 - (1.0*0.5)] = 2.80). We excluded such results, as they would not be possible in a real patient cohort.

### Quantifying maximum bias in an observational cohort

In order to determine the extent bias might have impacted a real cohort of DR-TB patients, we used data from the endTB observational cohort (ClinicalTrials.gov record NCT02754765). The endTB observational cohort is a prospective cohort of patients treated with bedaquiline and/or delamanid in 17 countries. Patients were eligible if they initiated an endTB regimen between 04/01/2015-11/16/2018 [18, 19]. The endTB Observational Study protocol was approved by central ethics review committees for each consortium partner, and local ethical approval was obtained in all endTB countries. All study activities were carried out following the principles of the Declaration of Helsinki. Participants provided written informed consent for inclusion in the observational cohort.

#### Baseline culture definitions

In primary analyses, we compared a baseline culture definition with an allowable interval of 90 days before (-90) and 0 days after (+0) treatment initiation to definitions extending the interval to 30, 60, and 90 days after treatment initiation. For the latter definitions, patients with any positive culture(s) in the allowable interval were considered to have a positive baseline culture. Patients without any cultures in the allowable interval were classified as missing a baseline culture.

#### Culture conversion definitions

We used an interim endpoint of six-month culture conversion, defined as two consecutive negative cultures collected at least 15 days apart, the first occurring up to 180 days after treatment initiation and the second up to 210 days after treatment initiation. Negative cultures were counted only if they were performed on samples collected after the baseline positive culture had been established. Participants who died or were LTFU before conversion were considered not to have converted because they had not experienced conversion and these events are considered unfavorable final treatment outcomes [20]. Death was due to any cause and LTFU was defined as treatment interruption for ≥2 consecutive months.

#### Calculating maximum bias in the frequency of culture conversion

We quantified the reported proportion with conversion (abbreviated C│P) as follows: $\frac{N\;Converted}{{N\;Culture\;positive}_{observed}}$. For conversion definitions including an interval extending past treatment initiation, we report the number of participants added and the number of participants missing a baseline culture who died or were LTFU during the post-treatment initiation interval. In order to investigate the upper bound of bias, we calculated the proportion converted, assuming a scenario of maximum bias (Converted│Culture positive_maximum bias,_ abbreviated C│P_mb_) as follows: $\frac{N\;Converted}{N\;{Culture\;positive}_{observed}\;+\;N\;died\;or\;LTFU│{Culture\;missing}_{observed}}$.

This equation reflects maximum bias in that it presumes 100% of patients with a missing pre-treatment culture who died or were LTFU during the allowable post-treatment initiation culture interval would have been observed to be culture-positive, had they had a culture. As this percentage decreases from 100% the expected magnitude of bias decreases.

## Results

### Quantifying bias using simulated data

**Fig 1:**
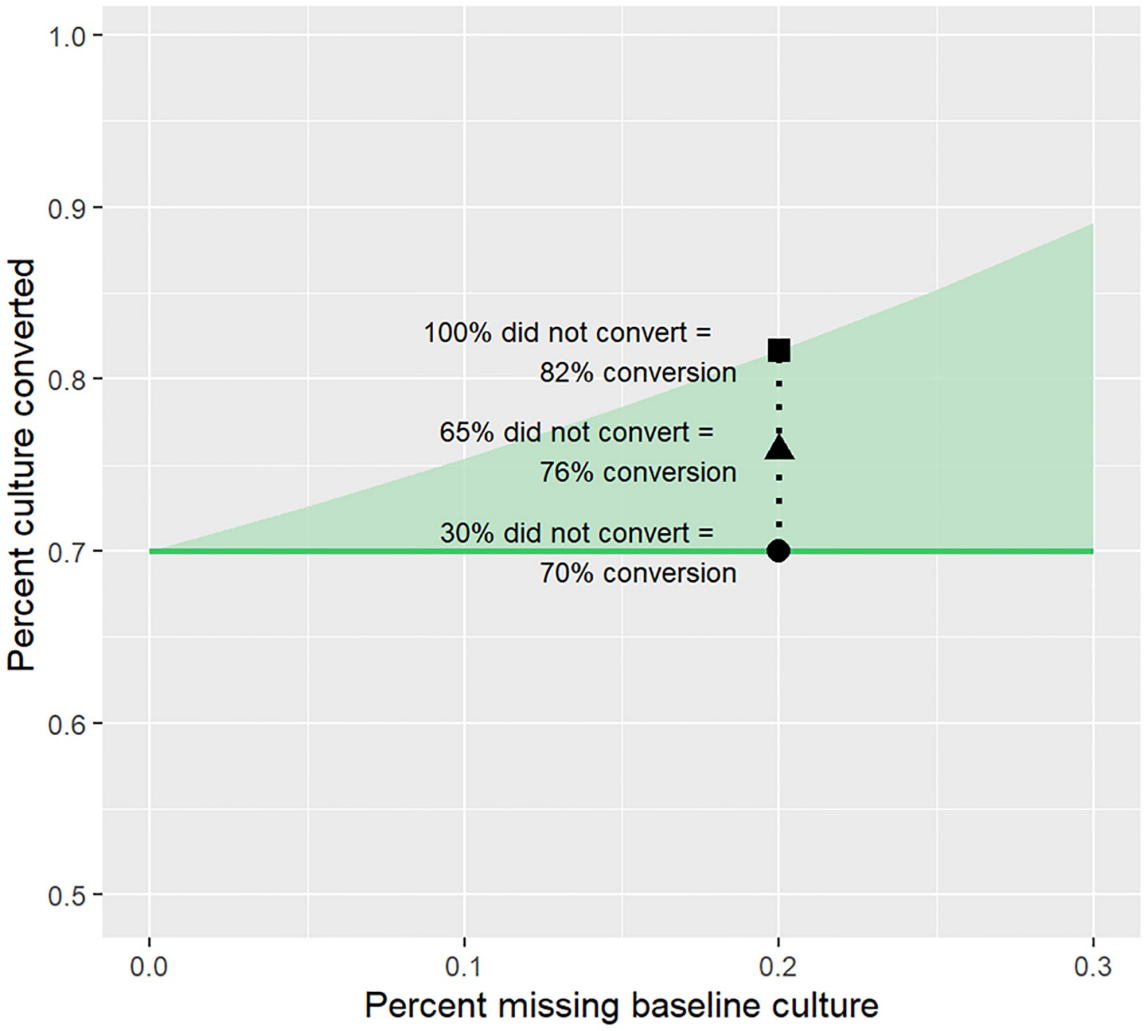
Culture conversion among participants missing a pre-treatment culture, simulation study of cohort where P_t_ = 70%, P_t_│m = 50%, and C│P_t_ = 70%. Fig 1 represents a simulated cohort of patients in which: 1) 70% are truly culture positive, 2) Of patients missing a culture 50% are truly culture positive and 50% are truly culture negative patients, and 3) among truly culture-positive patients, 70% achieved culture conversion. If 20% (x-axis = 0.20) of patients were missing their pre-treatment culture and 100% of these patients did not convert (e.g., due to early death or LTFU during the post-treatment interval), the observed proportion with culture conversion would be 82% (■), a 12 percentage point discrepancy. If 65% (i.e. the halfway point of the shaded region) did not convert, the observed proportion would be 76% (▲), a 6 percentage point discrepancy. If 30% did not convert (i.e. the point at which conversion frequencies in patients missing a baseline culture and patients observed to have a baseline culture are equal), the observed proportion would be 70% (●), no discrepancy. The shaded region can be interpreted similarly in Figs 2–4. **Abbreviations:** Culture positive_truth_ (P_t_); Culture positive_truth_│Culture missing_observed_ (P_t_│m); Converted│Culture positive_truth_ (C│P_t_).

**Fig 2:**
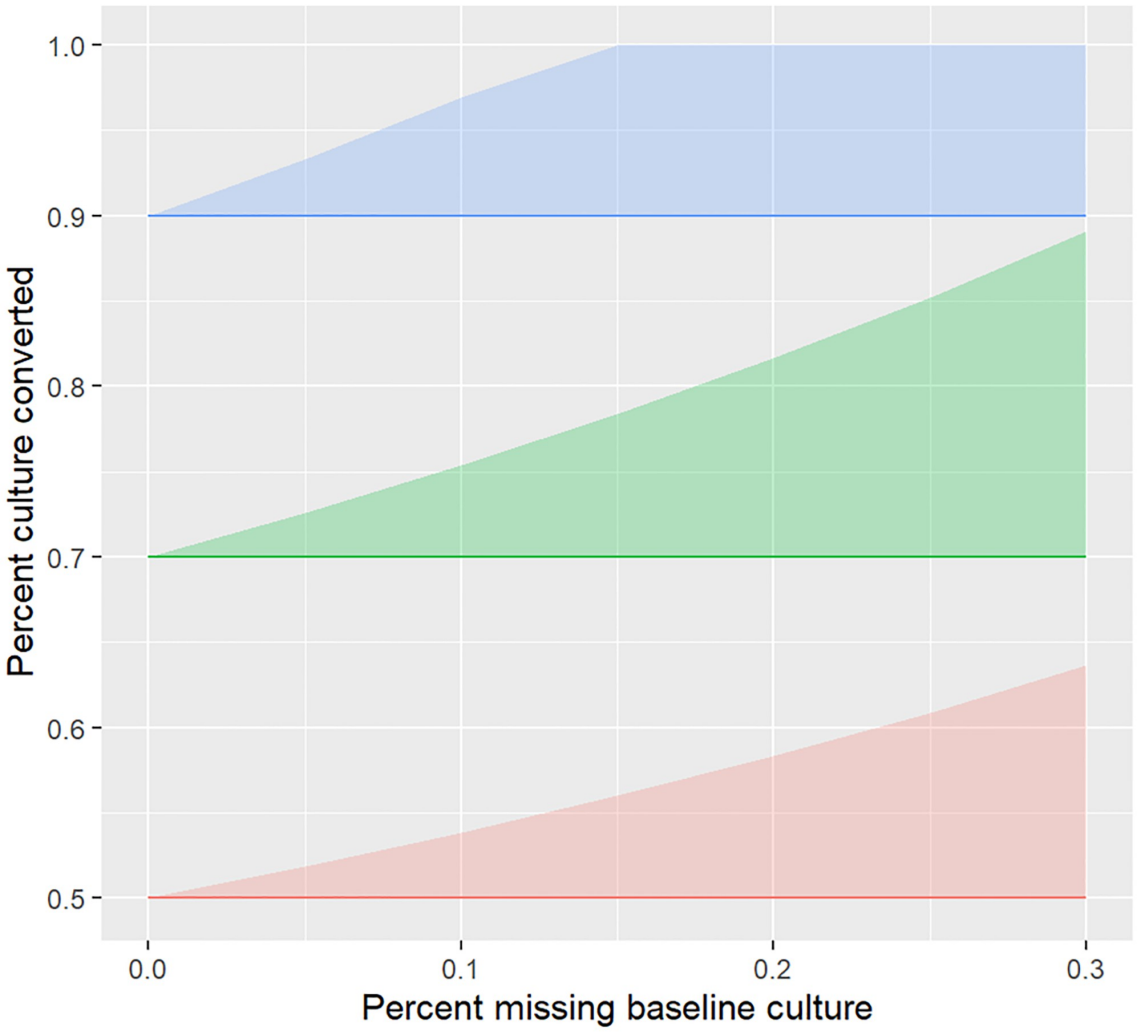
Culture conversion among participants missing a pre-treatment culture, simulation study of cohort where P_t_ = 70%, P_t_│m = 50%, and C│P_t_ = 50%, 70%, or 90%. In cohorts with high culture conversion frequencies (e.g. 90% conversion in blue), there are a finite number of patients who can be missing a pre-treatment culture (e.g. 15% at 90% conversion), assuming missing a pre-treatment culture is perfectly correlated with non-conversion due to early death or LTFU during the post-treatment interval (upper bound of shaded region).

**Fig 3:**
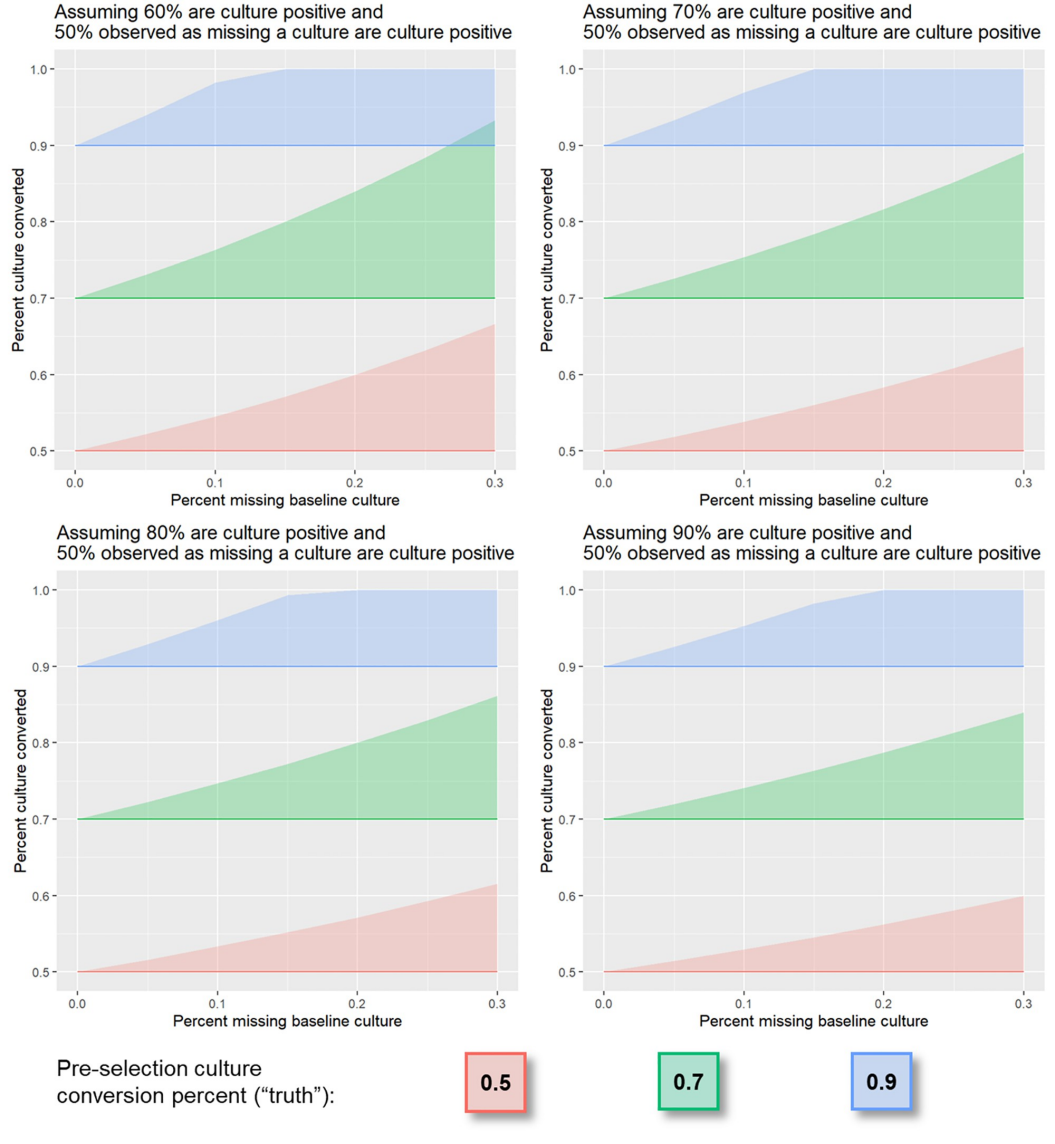
Culture conversion among participants missing a pre-treatment culture, simulation study of cohort where P_t_ varies from 60% to 90%, P_t_│m = 50%, and C│P_t_ = 50%, 70%, or 90%. As the proportion of patients culture positive at treatment initiation increases (each panel), the potential magnitude of bias decreases (shaded regions become smaller). This is because, assuming the proportion missing a culture who are culture positive is held constant (here, 50%), the exclusion of the same number of patients from a smaller cohort (i.e. smaller proportion of culture positive patients = smaller denominator) is more influential on the observed proportion with culture conversion than in a larger cohort (i.e. larger proportion of culture positive patients = larger denominator).

**Fig 4:**
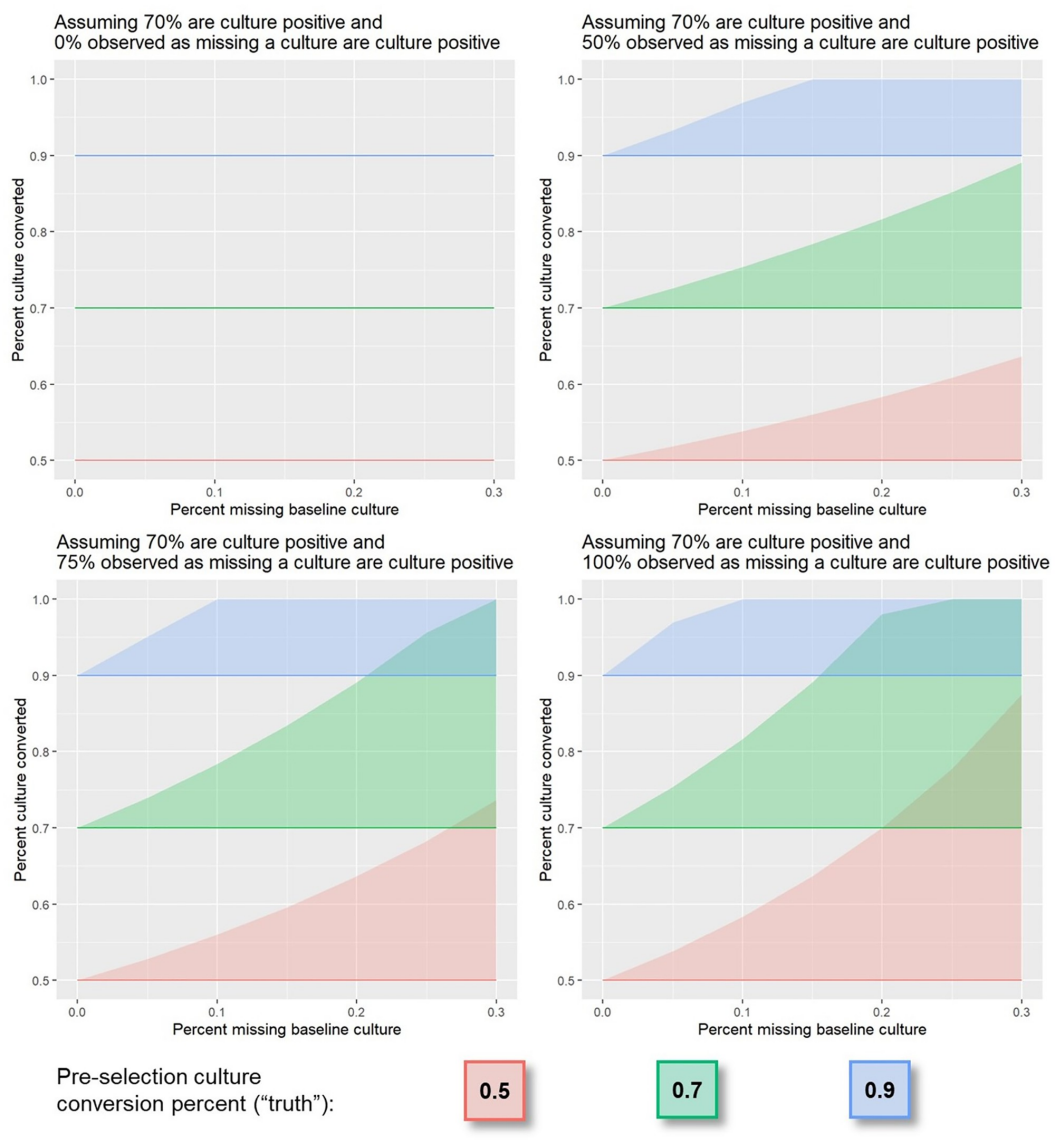
Culture conversion among participants missing a pre-treatment culture, simulation study of cohort where P_t_ = 70%, P_t_│m is varied from 0–100% and C│P_t_ = 50%, 70%, or 90%. The maximum magnitude of bias (top of each shaded region) is dependent on the proportion of patients who are culture positive among those missing a pre-treatment culture (each panel). If no (0%) patients missing a pre-treatment culture are culture positive, then these patients would be excluded from the analysis and no bias will be introduced (top left). Conversely, if all (100%) patients missing a pre-treatment culture are culture positive, then these patients should be included and the most potential for bias introduced (bottom right).

Combinations of the values listed in Table 1 resulted in 420 scenarios; 66 exceeded the 0.00 to 1.00 bounds for a proportion and were excluded, leaving 354 results. Fig 1 provides a guided interpretation of simulation results presented in Figs 2–4 and annotates two of the most influential drivers of bias. The horizontal line represents C│P_t_. The shaded region indicates the potential minimum and maximum bias as a function of the proportion of patients who did not convert among those missing a pre-treatment culture at the value of *m* on the x-axis. Thus, the upper bound of the shaded region represents the proportion observed if no patients missing a pre-treatment culture converted. The lower bound of the shaded region reflects the point conversion frequencies in patients missing a pre-treatment culture and patients observed to have a pre-treatment culture were the same. The two most influential drivers of bias, as shown by the width of the shaded region, are the proportion of patients missing a pre-treatment culture (x-axis) and the proportion of these patients who did not convert among those missing a pre-treatment culture (vertical point within shaded region), the latter of which we hypothesize to be due with death and LTFU during the post-treatment initiation interval.

Four patterns emerged from the simulation. First, the potential for bias increases as *m* increases, as shown by the larger width of shaded regions at higher values of the x-axis in Figs 2–4. Second, the potential for bias is limited in cohorts with high frequencies of conversion, as shown by the leveling off of the shaded region’s upper bound at 100% where C│P_t_ = 90% and m = ~15% in Fig 2. This is because the upper bound reflects a scenario in which all patients missing a pre-treatment culture do not convert and, in cohorts with high frequencies of conversion, there are a smaller number of patients who do not convert. Third, holding other parameters fixed, as P_t_ increases from 60% to 90%, the potential maximum bias decreases, as shown by the decreasing shaded regions’ widths across panels in Fig 3. This is because the sample size for a cohort in which 90% of patients are eligible (e.g. a denominator of 900 in a cohort of 1000 patients) will be larger than that in which 60% are eligible (e.g. a denominator of 600 in a cohort of 1000 patients). In addition, excluding the same number of patients from a cohort with a smaller denominator is more influential on the observed proportion than in a cohort with a larger denominator. Lastly, the magnitude of bias is dependent on P_t_│m (Fig 4). If all patients missing a pre-treatment culture would be culture negative had they had a culture, these patients would be excluded from analyses of conversion endpoints and no bias will be introduced. Conversely, if all patients missing a pre-treatment culture would be culture positive had they had a culture, these patients should be included. The magnitude of bias will then depend on the amount of missingness and non-conversion frequencies in this subset of the cohort, and to a lesser extent, the proportion of patients culture positive at treatment initiation, had they had a culture (Fig 3).

### Quantifying maximum bias in the endTB observational cohort data

In the endTB observational study, 2789 participants initiated a regimen and consented to participate. Of these, 1769 participants had a positive pre-treatment culture within 90 days before treatment initiation (-90/+0) and a 6-month conversion outcome (Table 2). Approximately 86% (N = 1518/1769) achieved conversion by 6 months. Applying a baseline culture definition extending 30 days past treatment initiation included 114 additional patients and yielded a similar conversion frequency (1614/1883, 86%).

**Table 2:** Sputum culture conversion and early death and loss-to-follow up events among participants missing a sputum culture in the specified interval before (-) and after (+) treatment initiation, endTB observational cohort.

Country	-90/+0 days	-90/+30 days	
	C│P *, n/N (%)	C│P *, n/N (%)	Died or LTFU, 1–30 days│m, N
Armenia	56/86 (0.65)	56/89 (0.63)	1
Bangladesh	182/187 (0.97)	189/194 (0.97)	0
Belarus	60/73 (0.82)	74/88 (0.84)	0
Ethiopia	29/34 (0.85)	33/39 (0.85)	0
Georgia	188/214 (0.88)	195/221 (0.88)	0
Haiti	16/24 (0.67)	17/25 (0.68)	0
Indonesia	27/40 (0.68)	33/48 (0.69)	4
Kazakhstan	400/418 (0.96)	414/433 (0.96)	1
Kenya	1/3 (0.33)	2/4 (0.50)	0
Kyrgyzstan	10/13 (0.77)	12/15 (0.80)	0
Lesotho	90/127 (0.71)	108/150 (0.72)	8
Myanmar	14/16 (0.88)	15/17 (0.88)	0
North Korea^†^	42/77 (0.55)	49/87 (0.56)	2
Pakistan	207/246 (0.84)	209/249 (0.84)	1
Peru	146/158 (0.92)	153/166 (0.92)	0
South Africa	25/26 (0.96)	29/30 (0.97)	0
Vietnam	25/27 (0.93)	26/28 (0.93)	0
**Total**	1518/1769 (0.86)	1614/1883 (0.86)	17

**Abbreviations:** Lost to follow up (LTFU), *Culture missing*_*observed*,_ (m); Converted│Culture positive (C│P)

* Observed proportion of the cohort with sputum-culture conversion, Converted│Culture positive_observed_ $(\text{C}│{\text{P}}_{\text{O}})=\frac{N\;Converted}{{N\;Culture\;positive}_{observed}}$

^†^ N = 3 patients in North Korea do not have a 6-month culture outcome and are excluded from the analysis

**Fig 5:**
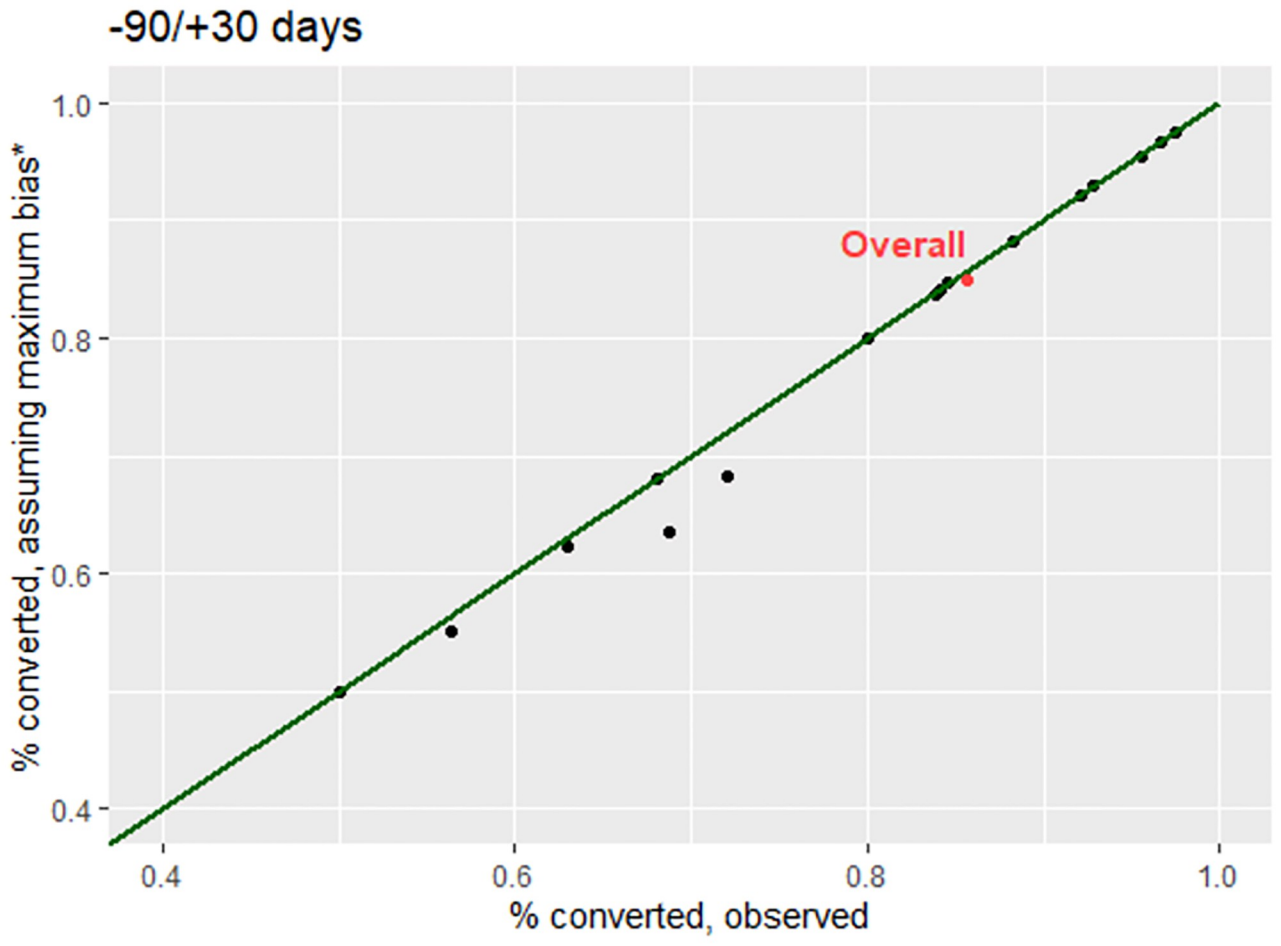
Absolute proportion of sputum culture conversion in the endTB observational cohort, by site assuming a baseline sputum culture collection interval 90 days before and after 30 days after treatment initiation. * Proportion of the cohort with sputum culture conversion, assuming maximum bias $\left(abbreviated\;{\%\;Converted│Cu+}_{\text{max}\;bias}\right)$ was calculated as follows: $\frac{N\;Converted}{N\;{Culture\;positive}_{observed}\;+\;N\;died\;or\;LTFU│{Culture\;missing}_{observed}}$. Sites on the green line indicate no deaths or loss-to-follow up events among participants with a missing culture occurred in the specified interval before (-) and after (+) treatment initiation.

Seventeen participants without a pre-treatment culture, nearly half of whom were treated in a single high HIV burden country, died or were LTFU in the 30-day period after treatment initiation. Assuming these participants would have been culture positive if they had had a culture, the proportion with conversion would be one percentage-point lower (85%, N = 1614/1900) than the observed proportion **(**Table 2, Fig 5, **-90/+30 days)**. The potential maximum bias differed by site. A 1–5 percentage point discrepancy between the observed proportion with conversion and proportion with conversion assuming maximum bias **(**Fig 5, **-90/+30 days)** was observed in the 6 sites with at least one death or LTFU event within 30 days of treatment initiation among participants missing a pre-treatment culture (Table 2). Similar findings were observed when extending the interval 60 and 90 days past treatment initiation (Supporting Information), with the exception of one site with a small sample size (N = 4 at -90/+90 days), which had a 10 percentage point discrepancy.

## Discussion

Using simulated data and observational cohort data, we investigated the potential for selection bias when extending baseline culture definitions to include a period after treatment initiation. Our findings have important implications for investigators reporting on descriptive outcomes of DR-TB treatment cohorts. In the present analyses, we identified the most influential factors increasing bias were the proportion of the cohort with a missing pre-treatment culture and the occurrence of death and LTFU in this group.

Simulation studies are a tool for exploring biases in epidemiology. However, simulations are only useful to the extent they are encoded with realistic values [16]. In simulations, we present the entire spectrum of bias in the reported proportion with conversion. In reality, the magnitude of bias is likely below the upper bound of these estimates because it is improbable that everyone missing a pre-treatment culture and who would have been culture positive had they had a culture does not convert (e.g., due to death or LTFU). Smaller amounts of potential bias suggests missing pre-treatment culture is less associated with non-conversion. Simulations with 20–30 percentage point overestimates of conversion proportions required a high frequency of culture-positive patients missing a pre-treatment culture (20–30%) and a high incidence of non-conversion (e.g., due to early death or LTFU in the post-treatment allowable interval) in this group. Early death rates of that magnitude are less common in today’s cohorts given advances in treatment have reduced mortality [21, 22], but not in historical cohorts. High early death rates were common among patients with advanced drug-resistance and HIV co-infection, such as in South Africa [23]. In fact, among DR-TB patients in South Africa between 2012–2014, 10% died within 12 weeks of treatment initiation and a missing or contaminated baseline culture was the strongest predictor of mortality [24]. While this study did not assess conversion, it is an example for which extending the allowable baseline interval past treatment initiation could introduce sizable bias. Theoretically, LTFU rates may have been higher in historical cohorts as well, given the toxicity of older regimens. Other context-dependent factors, such as the distance to treatment sites or war, could result in greater rates of early LTFU. Contamination may be an additional reason for missingness. Guidelines accept contamination frequencies ≤5% for fresh specimens and ≤10% for specimens requiring transport [25]. While contamination may increase the proportion of patients for whom a pre-treatment culture is missing, it is unlikely to be associated with either conversion or nonconversion, limiting the magnitude of bias.

We identified notable heterogeneity in bias across endTB sites. Site-specific differences provide insight into how this mechanism of selection bias can play out across settings with vastly different qualities of laboratory services, treatment programs, and comorbidities—features that our simulations did not explore. For example, high frequencies of early death and LTFU occurred in Lesotho, potentially due to more HIV and advanced drug resistance. And, in Kenya, a small number of early losses drastically biased conversion frequencies due to the site’s small size. Results from the simulation reinforce that low conversion frequencies and small proportions of patients with a positive pre-treatment culture increase bias, two common factors in high HIV burden cohorts. Investigators reporting on patients with comorbidities or other disease characteristics known to predict early death or LTFU should report whether these early events manifest in their cohort and the pre-treatment culture status of such patients. Several steps can be taken to prevent and assess the potential for this bias. During analysis, investigators could implement baseline culture definitions that do not extend the collection interval after treatment initiation. While this definition may exclude patients who had a culture shortly after treatment initiation, it also eliminates the potential for this source of bias. When investigators do extend the culture collection interval past treatment initiation, the investigator can simply check for death or LTFU events occurring in the post-treatment initiation interval among those without a pre-treatment culture. If these events occurred, the investigator should report them, as excluding these patients could introduce selection bias into the final estimate. Further, investigators could perform analyses to calculate the potential upper and lower bounds of the reported outcome to account for uncertainty due to possible selection bias or further explore the potential impact of selection bias by using inverse probability weighting [26, 27].

It is important to note that restricting the study sample to patients with a pre-treatment culture would exclude patients without a pre-treatment culture and could still impose selection bias. This source of selection bias is primarily an issue stemming from missing data and may be particularly problematic when using the interim endpoint of culture conversion if having a pre-treatment culture is associated with another variable. This source of bias is an inherent limitation of using culture conversion as an interim endpoint in the real-world setting where the likelihood of missing pre-treatment culture data is high and elucidates that an endpoint primarily developed for clinical trials may not transfer well to settings with less controlled clinical monitoring and data collection.

During data collection, investigators can avoid bias altogether by making dedicated efforts to collect sputum specimens before or on the day of treatment initiation. However, securing complete pre-treatment culture data is undoubtedly difficult in the context of observational DR-TB cohorts. Contamination occurs, patients may have difficulty producing sputum, and operational challenges to obtaining, processing, and transporting a specimen all impose barriers to complete data. Additionally, priority has been placed on decentralized capacity for rapid molecular diagnostics (e.g. Xpert^®^ MTB/RIF (Cepheid, Sunnyvale, USA)) [28]. This may reduce availability of (pre-treatment) cultures.

We present potential bias estimates disaggregated by each of the 17 enrollment sites of endTB to highlight heterogeneity that may arise from settings of different patient comorbidities and early treatment outcomes. In countries with small sample sizes, even a few patients with a missing pre-treatment culture who die or are LTFU can impose substantial bias in the proportion with conversion. Small sample sizes are not necessarily a study limitation, rather they reflect cohort sizes routinely reported on in the literature: 8% of MDR-TB cohort studies included less than 25 patients and 31% included less than 100 [6]. Second, in the endTB absolute proportion analysis, we calculate the proportion with conversion assuming maximum bias by adding to the denominator 17 patients who were missing a pre-treatment culture and died or were LTFU during the 30-day interval after treatment initiation. An additional 153 patients missing a pre-treatment culture were retained during this same period. We did not pursue analyses to assess how the exclusion of retained patients affected the observed proportion because doing so would require assumptions on the proportion who would have been culture positive had they had a culture and the conversion outcomes in this group, of which both assumptions lack previous evidence to inform their values. Thus, our endTB analysis effectively assumes retained patients with a missing culture would have converted at rates similar to patients with a culture.

## Conclusion

The implications of our study findings underscore the need to scrutinize whether bias is introduced when determining who is included and excluded from analyses with culture-based endpoints. Avoiding extension of the baseline culture interval past treatment initiation will eliminate the potential for bias. When this definition is extended past treatment initiation, the decision to do so should be reported and early death and LTFU events among excluded patients should be enumerated. Taking these steps will improve transparency and comparability of study findings across cohorts.

## Supporting information

S1 File (DOCX)

S1 Appendix (DOCX)

S1 Data (XLS)

## Abbreviations

C│P: Converted│Culture positive
C│P_mb_: Converted│Culture positive_maximum bias_
C│P_O_: Converted│Culture positive_observed_
C│P_t_: Converted│Culture positive_truth_
LTFU: loss to follow up
m: Culture missing_observed_
*M.tb*: *Mycobacterium tuberculosis*
MDR-TB: multidrug-resistant tuberculosis
P_t_: Culture positive_truth_
P_t_│m: Culture positive_truth_│Culture missing_observed_
RR: risk ratio
RR-TB: rifampin-resistant tuberculosis
TB: tuberculosis
WHO: World Health Organization
