# Supplementary material for: Selection bias in multidrug-resistant tuberculosis cohort studies assessing sputum culture conversion
Source: PLoS One. 2022 Nov 10;17(11):e0276457. doi: 10.1371/journal.pone.0276457 (PMC9648724; doi:10.1371/journal.pone.0276457)
Supplement: S1 File — (DOCX) [file pone.0276457.s001.docx]

**Supporting Information 1.** **Tree graph and guided example of quantification of bias through simulated data where *Culture positive_truth_* (P_t_)=70%, *Culture missing_observed_* (m)=20%, *Culture positive_truth_│Culture missing_observed_* (P_t_│m)=50%, and *Converted│Culture positive_truth_* (C│P_t_) = 70%**

**
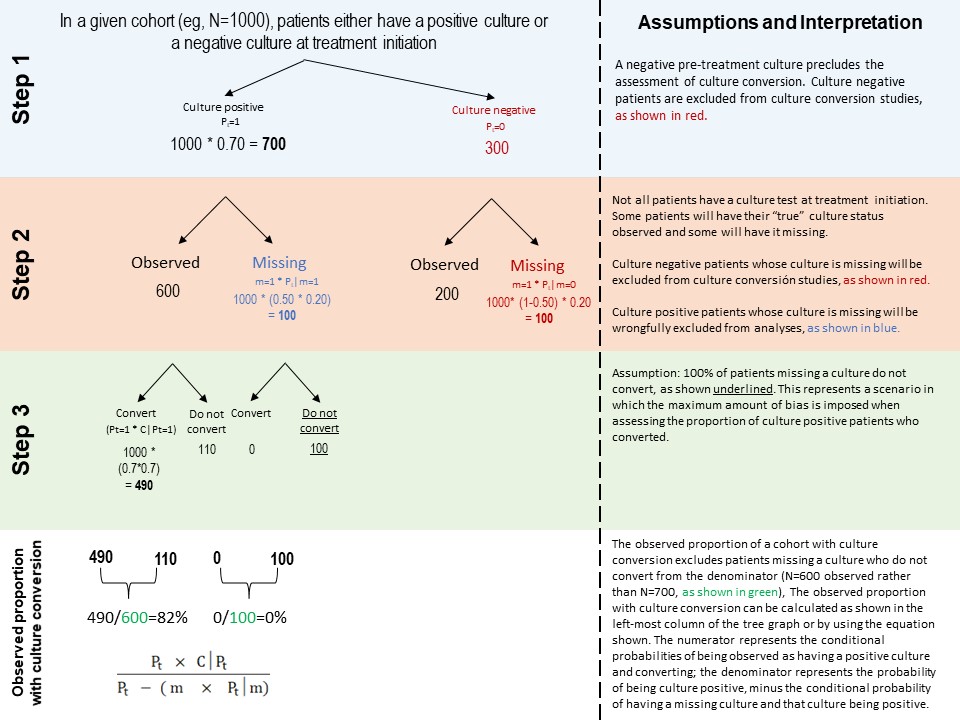
**

**Supporting Information 2.** **Sputum culture conversion and early death and loss-to-follow up events among participants missing a sputum culture in the specified interval before (-) and after (+) treatment initiation, endTB observational cohort**

| **Country** | **-90/+0 days** | **-90/+60 days** | | **-90/+90 days** | |
| --- | --- | --- | --- | --- | --- |
|  | C│P_O_ *,  n/N (%) | C│P_O_ *,  n/N (%) | Died or LTFU 1-60 days│m, N | C│P_O_ *,  n/N (%) | Died or LTFU 1-90 days│m, N |
| Armenia | 56/86 (0.65) | 56/89 (0.63) | 1 | 56/89 (0.63) | 1 |
| Bangladesh | 182/187 (0.97) | 192/197 (0.97) | 0 | 193/198 (0.97) | 0 |
| Belarus | 60/73 (0.82) | 82/96 (0.85) | 0 | 83/97 (0.86) | 0 |
| Ethiopia | 29/34 (0.85) | 33/39 (0.85) | 0 | 33/39 (0.85) | 0 |
| Georgia | 188/214 (0.88) | 198/225 (0.88) | 0 | 198/225 (0.88) | 0 |
| Haiti | 16/24 (0.67) | 17/26 (0.65) | 0 | 17/26 (0.65) | 0 |
| Indonesia | 27/40 (0.68) | 33/50 (0.66) | 4 | 33/51 (0.65) | 5 |
| Kazakhstan | 400/418 (0.96) | 419/440 (0.95) | 1 | 421/442 (0.95) | 1 |
| Kenya | 1/3 (0.33) | 2/4 (0.50) | 1 | 2/4 (0.50) | 1 |
| Kyrgyzstan | 10/13 (0.77) | 12/15 (0.80) | 0 | 12/15 (0.80) | 0 |
| Lesotho | 90/127 (0.71) | 111/155 (0.72) | 10 | 111/155 (0.72) | 10 |
| Myanmar | 14/16 (0.88) | 16/18 (0.89) | 1 | 17/19 (0.89) | 1 |
| North Korea^†^ | 42/77 (0.55) | 49/87 (0.56) | 5 | 49/87 (0.56) | 5 |
| Pakistan | 207/246 (0.84) | 210/250 (0.84) | 1 | 210/250 (0.84) | 2 |
| Peru | 146/158 (0.92) | 153/166 (0.92) | 0 | 153/166 (0.92) | 0 |
| South Africa | 25/26 (0.96) | 29/30 (0.97) | 0 | 30/31 (0.97) | 0 |
| Vietnam | 25/27 (0.93) | 26/28 (0.93) | 0 | 26/28 (0.93) | 0 |
| **Total** | 1518/1769 (0.86) | 1638/1915 (0.86) | 24 | 1644/1922 (0.86) | 26 |

**Abbreviations:** Lost to follow up (LTFU), *Culture missing_observed,_* (m); Converted│Culture positive_observed_ (C│P_O_)

***** Observed proportion of the cohort with sputum-culture conversion, Converted│Culture positive_observed_ (C│P_O_) _=_ $\frac{N Converted}{{N Culture positive}_{observed}}$

^†^ N=3 patients in North Korea do not have a 6-month culture outcome and are excluded from the analysis

**Absolute proportion of sputum culture conversion in the endTB observational cohort, by site and allowable baseline sputum culture collection interval before (-) and after (+) treatment initiation**


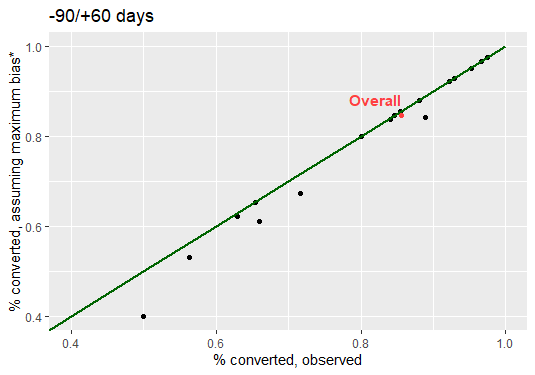

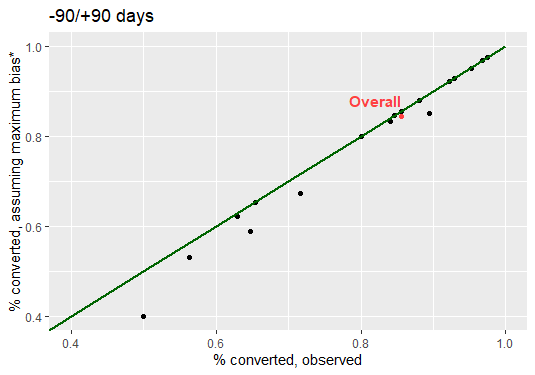


* Proportion of the cohort with sputum culture conversion, assuming maximum bias $({\% Converted│Cu+}_{\max bias})$ was calculated as follows: $\frac{N Converted}{N {Culture positive}_{observed} + N died or LTFU│{Culture missing}_{observed}}$.

**Legend:** Sites on the green line indicate no deaths or loss-to-follow up events among participants with a missing culture occurred in the specified interval before (-) and after (+) treatment initiation.

**Abbreviations:** Armenia (AM), Bangladesh (BD), Belarus (BY), Ethiopia (ET), Georgia (GE), Haiti (HT), Indonesia (ID), Kazakhstan (KZ), Kenya (KE), Kyrgyzstan (KG), Lesotho (LS), Myanmar (MM), North Korea (KP), Pakistan (PK), Peru (PE), South Africa (ZA), Vietnam (VD)
